# A unique life cycle transition in the red seaweed *Pyropia yezoensis* depends on apospory

**DOI:** 10.1038/s42003-019-0549-5

**Published:** 2019-08-07

**Authors:** Koji Mikami, Chengze Li, Ryunosuke Irie, Yoichiro Hama

**Affiliations:** 1 Faculty of Fisheries Sciences, Hokkaido University, 3-1-1 Minato-cho, Hakodate, 041-8611 Japan; 2 Graduate School of Fisheries Sciences, Hokkaido University, 3-1-1 Minato-cho, Hakodate, 041-8611 Japan; 3 Faculty of Agriculture, Saga University, 1 Honjo, Saga, 840-8502 Japan

**Keywords:** Plant physiology, Reproductive biology

## Abstract

Plant life cycles consist of two temporally separated stages: a haploid gametophyte and a diploid sporophyte. In plants employing a haploid–diploid sexual life cycle, the transition from sporophyte to gametophyte generally depends on meiosis. However, previous work has shown that in the red seaweed *Pyropia yezoensis*, this transition is independent of meiosis, though how and when it occurs is unknown. Here, we explored this question using transcriptomic profiling of *P*. *yezoensis* gametophytes, sporophytes, and conchosporangia parasitically produced on sporophytes. We identify a knotted-like homeobox gene that is predominately expressed in the conchosporangium and may determine its identity. We also find that spore-like single cells isolated from the conchosporangium develop directly into gametophytes, indicating that the gametophyte identity is established before the release of conchospores and prior to the onset of meiosis. Based on our findings, we propose a triphasic life cycle for *P*. *yezoensis* involving production of gametophytes by apospory.

## Abstract (web summary)

Koji Mikami et al. report transcriptome analysis of the haploid and diploid life stages of the red seaweed *Pyropia yezoensis*. They identify a homeobox gene that may be responsible for determining the identity of the conchosporangium and propose that this parasitic structure may represent a third life stage of the alga.

## Introduction

The life cycle in plants progresses by the repetitive appearance of haploid gametophyte and diploid sporophyte generations^1,2^. Since haploid and diploid genomes are created by meiosis and fertilization, respectively, switching of generations in the sexual life cycle is triggered by these events^2–6^. Due to differences in the temporal dominance of each generation, life cycle strategies are categorized into three major types: diploid, haploid, and haploid–diploid^3,4,6^. The diploid life cycle has a very short haploid gametophytic phase as single-celled male and female gametes, whereas in the haploid life cycle, meiosis occurs just after sexual gamete fusion; thus, the gametophytic phase becomes dominant. In the haploid–diploid life cycle, meiosis and fertilization are temporally separated, which results in mutually independent multicellularity and the concurrent existence of both gametophyte and sporophyte. The elucidation of the regulatory mechanisms controlling these life cycle strategies is essential for understanding fundamental biological issues, such as development and reproduction.

Regulatory mechanisms and master regulators of the generation transition in the sexual haploid–diploid life cycle have recently been analyzed using a reverse-genetic approach in the moss *Physcomitrella patens*, which belongs to Streptophyta, by production of genetic mutants that exhibit meiosis- and fertilization-independent generation switching. For instance, gene disruption of components of the polycomb repressive complex 2 (PRC2), like curly leaf and its associated partner fertilization independent endosperm, led to a transition from gametophyte to sporophyte without fertilization of sexual gametes^7,8^. PRC2 represses epigenetic switching to the sporophyte in the gametophytic generation via modification of the histone H3 K27me3^9^, which might be canceled by fertilization in the normal life cycle. In addition, overexpression of the three-amino-acid-length extension class homeodomain (TALE-HD) protein bell-like 1 (BELL1) and inactivation of another TALE-HD protein, the class 2 knotted1-like homeobox (KNOX2), resulted in transition of the life cycle generation without fertilization and meiosis, respectively^10,11^. Therefore, it has been proposed that the repression of *BELL1* gene expression by PRC2 is required for the maintenance of the gametophytic phase, and that maintenance of the sporophyte generation is performed by KNOX2, probably with BELL1 by heterodimerization. These findings demonstrate the critical involvement of meiosis and fertilization for the correct progress of the sexual life cycle via activation or repression of TALE-HD proteins^2,5^. However, to date, little is known about TALE-HD proteins in red seaweeds of the order Bangiales.

The marine Rhodophyta *Pyropia yezoensis* exhibits a haploid–diploid sexual life cycle in which macroscopic leafy gametophytes (thallus) and microscopic filamentous sporophytes (conchocelis) appear alternately^12–14^. Since male and female gametes develop on thalli and fertilization of them establishes diploid carpospores from which conchocelis are produced, fertilization triggers the transition from gametophyte to sporophyte in *P*. *yezoensis* as is in other multicellular organisms. Once conchocelis grow, conchosporangia are parasitically produced on conchocelis and mature to release conchospores that develop into gametophytic thalli^13,14^. However, meiosis occurs within the first two cell divisions in *P*. *yezoensis*^15–17^ or at the first cell division^18,19^ in conchospore germlings. The same findings have been reported for other Bangiales^20–24^.

**Fig. 1:**
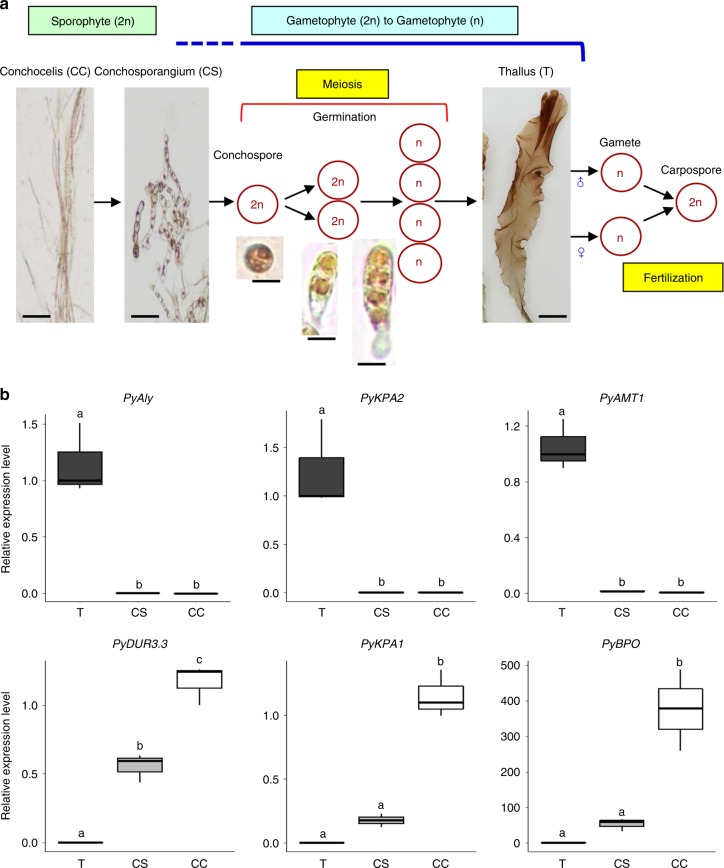
Temporal differences between establishment of gametophytic identity and meiosis in the heteromorphic haploid–diploid life cycle of *P*. *yezoensis*. **a** In the marine red seaweed *P*. *yezoensis*, the development of the thallus (T) starts from a conchospore that is released from the conchosporangium (CS) parasitically produced on the conchocelis (CC). Since conchospores develop into thalli and meiosis occurs during germination of conchospores, the transition from sporophyte to gametophyte is not associated with meiosis. By contrast, fertilization of male and female gametes is linked with the gametophyte–sporophyte transition in *P*. *yezoensis* as is observed in other eukaryotes. The figure is a modification of Fig. 1 in Shimizu et al.^9^. Scale bars: conchocelis and conchosporangium, 50 μm; thallus, 0.5 cm; conchospore and its germinating organisms, 15 μm. **b** Expression of previously reported thallus- and conchocelis-specific genes in the conchosporangium was examined by qRT-PCR. In box plots, values on the *Y* axis represent the fold change of relative quantification of each gene in T, CS, or CC. The *18S* rRNA transcript was quantified as an internal reference. Letters denote significant differences in expression level among the three life cycle stages from triplicate independent replicates as defined by the Tukey test (*p* < 0.05) in one-way ANOVA

Accordingly, it seems that the transition from sporophyte to gametophyte is independent of meiosis and that gametophyte identity is established before meiosis occurs in Bangiales, as depicted in Fig. 1a. This is inconsistent with the generally accepted concept of meiosis-dependent phase transition in eukaryotic organisms^2–5^, and little attention has been paid to this fact despite a long history of red seaweed research. It is unknown when and where sporophytes transit to gametophytes in the unique life cycle of *P*. *yezoensis* and other Bangiales. Here, we hypothesized that the conchosporangium could be the stage responsible for the generation transition from sporophyte to gametophyte.

To test this hypothesis, we examined the expression patterns of gametophyte- and sporophyte-specific genes in conchosporangia and performed comparative de novo transcriptome analysis to compare the genome-wide gene expression among the three life cycle stages, thalli, conchosporangia, and conchocelis, of *P*. *yezoensis*. In addition, we performed identification, phylogenetic classification, and expression analysis of *P*. *yezoensis* TALE-HD protein genes. We also investigated the generation identity of spore-like single cells forcibly released from the conchosporangium before the natural release of conchospores. Our results indicated the independence of conchosporangium as a life cycle generation and the critical involvement of conchosporangia in the generation switch from sporophyte to gametophyte in a meiosis-independent manner in *P*. *yezoensis*.

## Results

### Unique gene expression profiles in conchosporangia

Presuming that the gametophyte identity is established in the conchosporangium, we predicted that gametophyte-specific genes would be expressed during this life cycle stage. Therefore, we first evaluated the expression of thallus- and conchocelis-specific genes in the conchosporangium by quantitative reverse-transcriptase PCR (qRT-PCR). Previous reports have shown that genes encoding the alginate lyase (PyAly)^25^, sodium pump (PyKPA2)^26^, and ammonium transporter (PyAMT1)^27^ are thallus specific, whereas genes encoding the sodium pump (PyKPA1)^28^, bromoperoxidase (PyBPO)^29^, and urea transporter (PyDUR3.3)^30^ are conchocelis specific. Thus, we compared the expression levels of these genes in the thallus, conchosporangium, and conchocelis by qRT-PCR with gene-specific primers (Supplementary Table 1). Expression of the three thallus-specific genes was not detected in the conchosporangium nor in the conchocelis (Fig. 1b), indicating their restricted expression within the life cycle. In addition, conchocelis specificity of the expression of *PyKPA1* and *PyBPO* genes was confirmed (Fig. 1b). By contrast, the expression of the *PyDUR3*.*3* gene in the conchosporangium was significantly higher than that in the thallus (*p* < 0.05, one-way ANOVA) but less than its expression in the conchocelis (Fig. 1b), indicating that this is a conchocelis-biased gene. Therefore, we suggest that the conchosporangium is a unique life cycle stage in which known thallus- and conchocelis-specific genes are either not expressed or expressed at low levels.

### De novo transcriptome assembly and annotation

To gain further information about gene expression in the three different life cycle stages, we performed comparative de novo transcriptome assembly and analysis. Total RNA samples prepared from the thallus, the conchocellis, and the conchosporangium were separately used to construct three cDNA libraries that were sequenced using an Illumina HiSeq 4000 system. A total of about 13.57 GB was generated from the three life cycle stages; 4.56, 4.5, and 4.51 GB from the thallus, the conchocelis, and the conchosporangium cDNAs, respectively. After filtering of the reads, de novo assembly with clean reads was performed by the Trinity program, which resulted in 48,951 unigenes with a total length, an average length, N50, and GC content of 35,105,458 bp, 717 bp, 1,164 bp, and 66.57%, respectively. The size distribution for these unigenes is represented in Supplementary Fig. 1, indicating that 27,801 unigenes (56.8%) were >500 bp, and 16,786 unigenes (34.3%) were >1 kb.

Accurate annotation information of all unigenes was assigned by interrogation using seven functional databases, the NCBI nonredundant protein sequences (NR), NCBI nonredundant nucleotide sequences (NT), Swissprot, clusters of orthologous groups of proteins (COG), Kyoto Encyclopedia of Genes and Genomes database (KEGG), gene ontology (GO), and Interpro. Overall functional annotation (Supplementary Fig. 2a) indicated that 34,525 unigenes of a total 48,951 (70.53%) were successfully annotated in NR for 32,125 unigenes (65.63%), in NT for 12,461 unigenes (25.46%), in Swissprot for 26,629 unigenes (54.4%), in COG for 20,830 unigenes (42.55%), in KEGG for 28,683 unigenes (58.6%), in GO for 3,487 unigenes (7.12%), or in Interpro for 15,157 unigenes (30.96%). In addition, NR annotation revealed that the distribution for annotated species were *Chondrus crispus* (11.58%), *Aureococcus anophagefferens* (8.54%), *Emiliania huxleyi* CCMP1516 (6.76%), and *Guillardia theta* CCMP2712 (4.97%), although 68.15% of all unigenes showed no homology to annotated species (Supplementary Fig. 2b).

A total of 42.55% of the unigenes were annotated using COG based on sequence homology and were classified into 25 functional classifications, in which the dominant terms were “translation, ribosomal structure, and biogenesis” (7856 unigenes) and “cell cycle control, cell division, and chromosome partitioning” (6483 unigenes) (Supplementary Fig. 3). “Cell wall/membrane/envelop biosynthesis,” “carbohydrate transport and metabolism,” and “replication, recombination, and repair” also shared a high percentage of genes among the categories (Supplementary Fig. 3). In addition, all unigenes were classified into 45 functional items by annotating with a GO assignment. A large percentage of genes was associated with the molecular function categories (catalytic activity and binding), the cellular component (cellular process, cell, cell part, and organelle), and biological process (cellular process and metabolic process) (Supplementary Fig. 4). Moreover, all unigenes were classified to 21 functional pathways based on the KEGG database (Supplementary Fig. 5). The metabolic pathways were dominant (11,133 unigenes), with most unigenes involved in “global and overview map” (4583 unigenes). “Translation” and “transport and catabolism” also shared numerous genes among the categories with 6126 and 3476 unigenes, respectively.

### Unique gene expression profiles in the conchosporangium

**Fig. 2:**
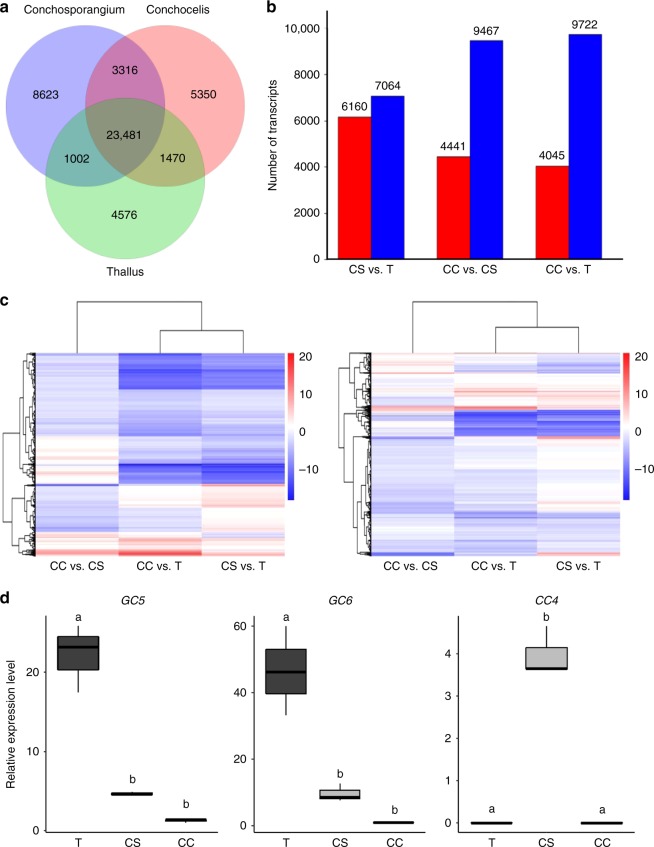
Transcriptional changes among three different life cycle stages. **a** Venn diagram displaying the number of unique and overlapping unigenes expressed in the thallus (green), conchosporangium (blue), and conchocelis (pink). **b** Comparisons of numbers of differentially upregulated (red bars) and downregulated (blue bars) unigenes between three combinations of three life cycle stages (CS vs. T, CC vs. CS, and CC vs. T). T, thallus; CS, conchosporangium; CC, conchocelis. **c** Heatmap representation of a cluster analysis of the gene expression patterns of the DEGs among three life cycle stages. The FPKM change patterns of three combinations of two life cycle stages (CC vs. CS, CC vs. T, and CS vs. T) were clustered. Left panel, analysis for intersection of sets; right panel, analysis for union of sets. **d** Box plots showing quantitative RT-PCR validation analysis of expression of selected thallus-biased (*GC5* and *GC6*) and conchosporangium-specific (*CC4*) unigenes. The ratio values on the *Y* axis refer to the relative expression levels of genes selected from Supplementary Table 2 among three life cycle stages, T, CS, and CC. The *18S* rRNA transcript was quantified as an internal reference. Letters denote significant differences in expression level among three life cycle stages from triplicated experiments as defined by the Tukey test (*p* < 0.05) in one-way ANOVA

We used the de novo transcriptome assembly to gain further insights into the expression profiles of the different life stages. A total of 48,951 differentially expressed genes (DEGs) were identified (Fig. 2a). In the conchosporangium, there were 6160 and 4441 upregulated DEGs compared with the thallus and conchocelis, respectively, and the corresponding numbers of downregulated DEGs were 7064 and 9467 (Fig. 2b). In addition, the conchocelis had 4045 and 9722 upregulated and downregulated DEGs, respectively, compared with the thallus, which is similar to the number of DEGs in the conchocelis vs. the conchosporangium (Fig. 2b). Thus, the total gene expression profiles in the thallus and conchosporangium were quantitatively similar. Figure 2c shows a heatmap representation of a cluster analysis of the expression patterns of DEGs among the three life cycle stages. These results indicate that although the overall gene expression pattern was similar between the conchocelis and the conchosporangium, upregulated and downregulated genes in the thallus were qualitatively different from those in the conchocelis and conchosporangium. Indeed, as shown in Supplementary Table 2, in which the expression of the unigenes is represented by comparative fragments per kilobase per million (FPKM) values among the three life cycle stages, there were stage-specific genes as well as thallus- and conchocelis-biased genes whose expression was also found in the conchosporangium.

To validate the reliability of the differential gene expression revealed by the comparative transcriptome analysis, qRT-PCR was performed for nine DEGs selected according to their expression profiles (Supplementary Table 2) with primer sets specific to each unigene (Supplementary Table 3). Fold change values of differential expression for each DEG obtained by the qRT-PCR analysis are displayed in Fig. 2d. The conchosporangium specificity of Unigene10116 (*CC4*) was confirmed, as its expression was detected in the conchosporangium only (Fig. 2d). Conchocelis-specific expression was also detected with the DEGs *SS3*, *SS4*, and *SS5*, albeit at a very low level (Supplementary Fig. 6). In addition to these stage-specific genes, the expression levels of thallus-dominant DEGs such as *GC5* and *GC6* were higher in the conchosporangium than in the conchocelis (Fig. 2d). Thus, the expression profiles of these selected DEGs were consistent with the results of the transcriptome analysis (Supplementary Table 2), implying that the qRT-PCR results are credible. These results of the qRT-PCR analysis clearly indicate that conchosporangia exhibit unique gene expression profiles relative to the other life cycle stages tested.

### Expression of a TALE-HD gene during the life cycle

**Fig. 3:**
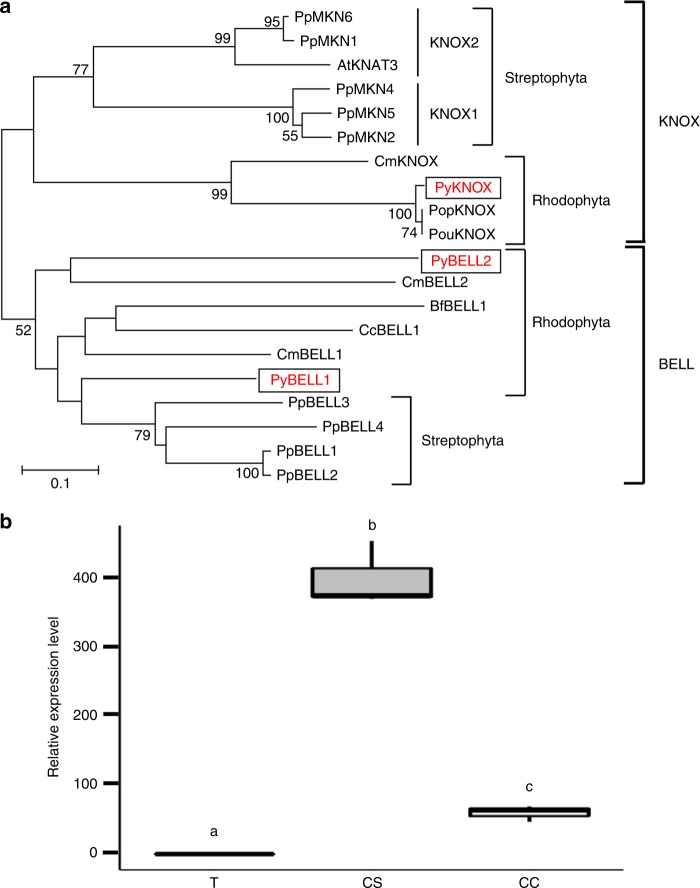
Conchosporangium-predominant expression of the *PyKNOX* gene during the life cycle of *P*. *yezoensis*. **a** Phylogenetic classification of three TALE homeobox genes from *P*. *yezoensis*. Amino acid sequences of the conserved TALE-HD domains from Bangiales and the land green plants *Arabidopsis thaliana* and *P*. *patens* were used for construction of neighbor-joining-based unrooted phylogenetic tree with the ClustalW and MEGA 8.0 programs. The bootstrap values with 1000 replicates over 50% are indicated at the nodes of the tree. The TALE homeobox proteins from *P*. *yezoensis* are boxed. Accession numbers: *P*. *patens*, PpMKN1 BAF285148, PpMKN2 BAF96739, PpMKN4 BAF96740, PpMKN5 BAF96741, PpMKN6 XM_001765523, PpBELL1 XP_001779432, PpBELL2 XP_001777380, PpBELL3 XM_001769443, PpBELL4 XP_001762111; *Arabidopsis thaliana*, AtKNAT3 X92392; *Chondrus crispus*, CcBELL1 XP_005711328; *Cyanidioschyzon merolae*, CmKNOX (CMR153C) XP_005538442, CmBELL1 (CMH049C) XP_005536034, CmBELL2 (CMR176C) XP_005538457. PopKNOX (esisotig02479) and PouKNOX (esisotig04347) are KNOX homologs of *Porphyra purpurea* and *Porphyra* *umbilicalis*, respectively, which were derived from NoriBLAST (http://dbdata.rutgers.edu/nori/). Gene IDs derived from our transcriptome analysis: *P*. *yezoensis*, PyKNOX CL1448 (Accession no. MK629536), PyBELL1 CL1176 (Accession no. MN070241), PyBELL2 Unigene19722 (Accession no. MN070242); *Bangia fuscopurpurea*, BfBELL1 Unigene3027. **b** Box plots showing qPCR validation of *PyKNOX* gene expression. The ratio on the *Y* axis refers to the relative expression levels of the *PyKNOX* gene among three life cycle stages, thallus (T), conchosporangia (CS) and conchocelis (CC). The *18S* rRNA transcript was quantified as an internal reference. Letters denote significant differences in expression levels among the three life cycle stages from triplicated experiments as defined by the Tukey test (*p* < 0.05) in one-way ANOVA

TALE-HD proteins like BELL1 and KNOX2 are master regulators of the life cycle in the moss *Physcomitrella patens*^2,5,10,11^. We investigated whether genes encoding TALE-HD proteins are also expressed in a generation-specific manner during the life cycle of *P*. *yezoensis*. First, since our transcriptome analysis revealed the presence of at least three TALE-HD protein genes (Contigs CL1448 and CL1176, Unigene19722), we performed a phylogenetic analysis to classify these genes. This showed that CL1448 clustered in the KNOX family; however, genes from Rhodophyta formed a sister clade that was distantly related to both the KNOX1 and KNOX2 subfamilies. Thus, we designated the *CL1448* gene *PyKNOX* (Fig. 3a). CL1176 and Unigene19722 were categorized as BELL gene homologs and thus were designated *PyBELL1* and *PyBELL2*, respectively (Fig. 3a). *PyBELL1* was more closely related to the *BELL* genes from the moss than *PyBELL2*, which was located in a Rhodophyta *BELL* sister clade, distantly related to the Streptophyta *BELL* clade. Partial amino acid sequences of the TALE-HD proteins from *P*. *yezoensis*, including the conserved homeobox KN domain, are shown in Supplementary Fig. 7.

We then investigated the expression patterns of the genes encoding the *P*. *yezoensis* TALE-HD proteins during the *P*. *yezoensis* life cycle. We chose the *PyKNOX* gene to perform qRT-PCR analysis because the PyBELL contigs were too short to design primers for quantitative expression analysis. As shown in Fig. 3b, the *PyKNOX* gene was predominantly expressed in the conchosporangium, whereas very low expression was detected in the sporophyte (the conchocelis) and no expression was detected in the gametophyte (the thallus). Given that TALE-HD proteins are key determinants of identities of life cycle generations in moss plants^2,5,10,11^, we hypothesize that PyKNOX determines the conchosporangium identity, suggesting the uniqueness of this stage in the life cycle of *P*. *yezoensis*, which is consistent with the results in Fig. 2.

### Establishment of gametophytic identity in the conchosporangium

**Fig. 4:**
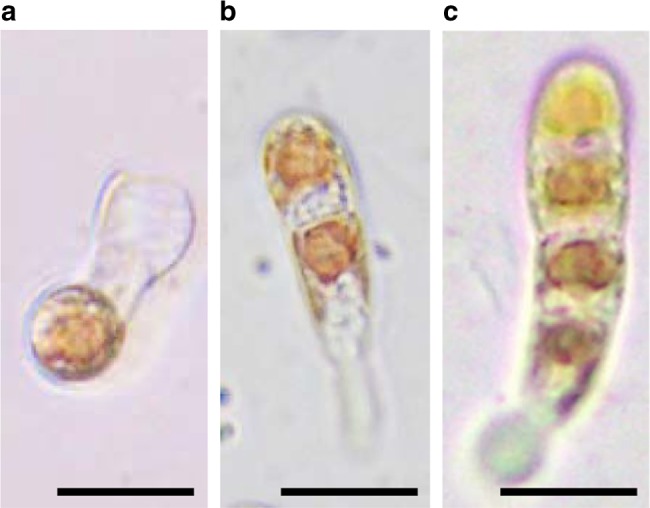
Spore-like single cells isolated from conchosporangia develop as gametophytes. Fragmented conchosporangia were isolated and cultured to observe early development. Representative images are shown. **a** Spore-like single cell just after release from the conchosporangium from the cell wall. After release, this cell moved autonomously before adhering to the base of the culture dish and thus became separated from the cell wall skeleton. **b**, **c** Two- and four-cell stages of the resulting germling, demonstrating the gametophytic development of the spore-like single cell from the conchosporangium. Scale bar: 20 μm

To address whether gametophyte generation truly occurs in conchosporangia, spore-like single cells, which were isolated artificially from the conchosporangium by chopping it with a razor blade into one to five cell fragments before the release of conchospores, were cultured to observe their early development. The efficiency of the isolation was low, but once spore-like single cells appeared, these cells developed into thalli with normal development and morphology (Fig. 4), whereas no filamentous conchocelis was produced. These findings indicate that gametophytic identity is established in the conchosporangium before the release of conchospores.

## Discussion

**Fig. 5:**
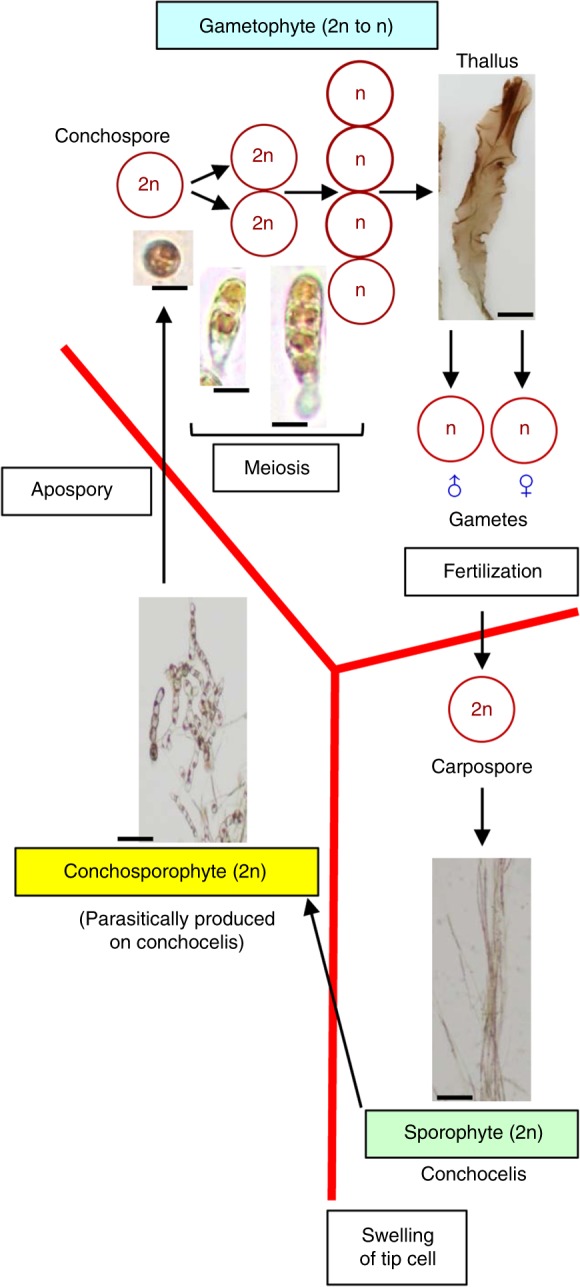
The triphasic hypothesis for the heteromorphic sexual life cycle of the Bangiales. We propose the presence of three different generations in the Bangiales life cycle and the renaming of the conchosporangium to the conchosporophyte, as an independent generation in the life cycle. The positions of the generation switches in the diphase concept are ambiguous, especially for the sporophyte–gametophyte transition (see Fig. 1). By contrast, the triphasic hypothesis clarifies the events of the generation transitions; the fertilization of male and female gametes for the gametophyte–sporophyte transition, the swelling of the conchocelis tip cell for the sporophyte–conchosporophyte transition, and apospory for the conchosporophyte–gametophyte transition that is independent of meiosis. Scale bars: sporophyte and conchosporophyte, 50 μm; thallus, 0.5 cm; conchospore and its germinating organisms, 15 μm

The discovery of the conchocelis as the sporophyte generation in *Porphyra umbilicalis*^31^ led to the theory that the Bangiales display a heteromorphic and diphasic sexual life cycle. Thus, the life cycle stage is defined as separate and free-living phases. In general, the conchocelis-parasitizing conchosporangium has been categorized as a sporophyte whose shape is different from the conchocelis. Here, however, transcriptome analysis revealed that the gene expression profile of the conchosporangium was different from that of the thallus and conchocelis. This is supported by the identification of conchosporangium-specific and thallus- and conchocelis-biased unigenes (Figs. 2 and 3, Supplementary Table 2). It has been accepted that the Florideophyceae class of red seaweeds displays a triphasic sexual life cycle, in which diploid carposporophytes are generated parasitically on female haploid gametophytes after fertilization of male and female gametes. The diploid carpospores are produced in a carposporophyte on female gametophytes and the released carpospores develop into diploid tetrasporophytes. In the tetrasporophyte, a number of tetrasporangia are generated to produce haploid tetraspores, via meiosis, as male or female gametes^32,33^. Thus, tetrasporophyte amplifies the reproductive production of gametophytic tetraspores. It is similar to conchosporangia that also amplify the production of conchospores with gametophytic identity (Fig. 1a), suggesting that the conchosporangium of the Bangiales is functionally the same as the tetrasporophyte of the Florideophyceae, although meiosis has not occurred in this stage (Fig. 1a). These findings indicate that the conchosporangium is a separate life cycle phase, different from the gametophytic thallus and sporophytic conchocelis, although the conchosporangium is not free living by its parasitical production on conchocelis filaments as the carposporophyte produced on female gametophytes in Florideophyceae. In addition, this identity was confirmed by qRT-PCR analysis that indicated the predominant expression of *PyKNOX* in the conchosporangium (Fig. 3). Based on these findings, we conclude that the conchosporangium is an independent generation in the life cycle of the Bangiales. Accordingly, we propose a triphasic heteromorphic sexual life cycle hypothesis for the Bangiales and should be renamed as the conchosporophyte (Fig. 5), where the conchosporangium bridges both the sporophytic and gametophytic generations.

Production of gametophytic conchospores in conchosporophyte is a representative characteristic in the proposed triphasic life cycle of *P*. *yezoensis*. Since spore-like single cells isolated from the conchosporangium developed into thalli (Fig. 4) and meiosis occurs during early germination of released conchospores in *P*. *yezoensis*^15–19^, gametophytic identity must be established in the conchosporophyte in a meiosis-independent manner before conchospore discharge. This is contrary to current thinking, in which meiosis plays a critical role in phase transitions during the eukaryotic haploid–diploid life cycle^2–6^. Phase transition from sporophyte to gametophyte without meiosis is known as apospory^34–36^. Together with observations indicating that meiosis occurs during conchospore germination in various *Pyropia* and *Porphyra* species^20–24^, we conclude that *P*. *yezoensis* employs apospory as a natural strategy for generation switching to generate gametophytes and the uncoupling of meiosis and generation transition by apospory is a common characteristic in the Bangiales.

It is not well known when the sporophyte–conchosporophyte transition occurs in the life cycle of the Bangiales. In this respect, it is worth noting that swelling of the tip cell of conchocelis branches is observed as an initial stage of the conchosporangium development and the swollen cell grows into a multicellular conchosporangium^37^, while branches that lack swelling grow continually as conchocelis. Thus, it is plausible that swelling triggers the sporophyte–conchosporophyte transition. Interestingly, the marine red seaweed *Pyropia tenuipedalis* lacks the conchosporophyte phase; thus, the tip cell of sporophyte branches directly develops into the gametophyte after swelling^38^, which strongly supports the importance of swelling of the tip cells for phase transition in the Bangiales. In fact, we observed tip growth of the conchocelis (Supplementary Fig. 8) and the conchosporangium^39^, where the tip cell continually produces two cells through asymmetric cell division: an apical tip cell with proliferating activity and a neighboring differentiated vegetative cell. Thus, we propose that the tip cells in these two stages are apical stem cells and the transition from sporophyte to conchosporophyte results in a change of identity of the conchocelis stem cell to the conchosporangium stem cell, which is probably stimulated by swelling of the tip cells of the conchocelis branches.

We demonstrated the presence of three TALE-HD genes in *P*. *yezoensis* and observed conchosporangium-predominant expression of the *PyKNOX* gene (Fig. 3). It has been demonstrated that inactivation of the *KNOX2* gene resulted in apospory in the moss *P*. *patens*^10^, indicating that *KNOX2* represses the expression of genes involved in the establishment and maintenance of gametophytic identity in sporophytes of terrestrial plants. Thus, it is possible that apospory in *P*. *yezoensis* requires an inhibition or loss of activity of PyKNOX involved in the maintenance of conchosporophyte identity. However, PyKNOX seems to be functionally different from KNOX2 because apospory in the Bangiales is implemented after the sporophyte–conchosporophyte transition. Thus, we postulate that PyKNOX is activated by the swelling of the apical tip cells of the conchocelis. Therefore, functional analyses of PyKNOX in the sporophyte–conchosporophyte transition, and of maintenance of the conchosporophyte stage, combined with investigation of the expression and functions of the *PyBELL* genes, are necessary to confirm our triphasic sexual life cycle hypothesis for the Bangiales.

## Methods

### Plant materials and cultivation conditions

The leafy gametophyte (thallus), conchosporangia, and filamentous sporophyte (conchocelis) of *P*. *yezoensis* strain U-51 were cultured separately in enriched sea life (ESL) medium^40^, which was made by dissolving commercially available Sealife powder (Marintech Co. Ltd, Japan) in distilled water with the addition of ESS_2_ solution, at 15 °C under irradiation with 60 μmol photons m^−2^ s^−1^ provided by cool white fluorescent lamps with a photocycle of 10 h light and 14 h dark. The medium was bubbled continuously with filter-sterilized air and changed weekly.

### Construction and sequence analysis of cDNA libraries

Construction of cDNA libraries and transcriptome sequencing was performed by BGI (Shenzhen, China) on HiSeq instruments (Illumina Inc., San Diego, USA) in accordance with the manufacturer’s instructions. Library construction was performed using the TruSeq RNA Sample Prep Kit v2 (Illumina Inc., San Diego, USA). First, total RNA was obtained separately from the gametophyte, conchosporangium, and sporophyte samples and then mRNA was purified from 200 ng total RNA with oligo-dT Beads after DNase I treatment. After mixing with fragment buffer, the poly(A)-containing mRNA was sheared into short fragments. Using these mRNA fragments as templates, first-strand cDNAs were generated by reverse transcription reactions with First Strand Master Mix and Super Script II (Invitrogen, Waltham, Massachusetts, USA) under the following conditions: 25 °C for 10 min, 42 °C for 50 min, and 70 °C for 15 min. Then, Second Standard Master Mix was added to synthesize the second-strand cDNA. The resulting fragmented cDNA was repaired with End Repair Mix for 30 min at 30 °C, purified with AMPure XP beads (Agencourt; Beckman Coulter, Brea, California, USA), and phosphorylated using A-Tailing Mix by incubation at 37 °C for 30 min. These short cDNA fragments were then connected to the RNA index adapter using Ligation Mix at 30 °C for 10 min and purified with AMPure XP beads. The purified cDNA fragments were amplified with PCR Primer Cocktail and PCR Master Mix to enrich the cDNA fragments. The final libraries were quantified and qualified by the Agilent 2100 bioanalyzer instrument (Agilent DNA 1000 Reagents) and real-time quantitative PCR (qPCR) with the TaqMan probe, respectively. These qualified libraries were amplified on the cBot system to generate clusters on a flowcell (TruSeq PE Cluster Kit V3-cBot-HS; Illumina) and sequenced using the Illumina HiSeq 4000 platform (Illumina). The average read length was 90 nucleotides.

### De novo assembly and transcriptome data analyses

Both de novo assembly and transcriptome data analyses were performed by BGI (Shenzhen, China). Strict filtering of the reads was performed before assembly. First, paired-end reads with primer or adapter sequences were removed. Then, reads with more than 5% of bases as unknown nucleotides and other low-quality reads were filtered from total reads to correct clean reads. Trinity v2.0.6^41^ software was applied for transcriptome de novo assembly and then the clean reads were mapped back to contigs by Tgicl v2.0.6^42^ to reduce redundancy and used for downstream analysis. The longest assembled sequences were defined as unigenes.

With functional annotation, extraction of coding sequences (CDs) was achieved from unigene sequences that were then translated into peptide sequences. CDs of unigenes without any BLAST hits were further predicted by ESTScan^43^ and were also translated into peptide sequences. The DEGs were determined by PossionDis according to Audic and Claverie^44^, and the expression level of each unigene was calculated using FPKM by RNASeq by expectation maximization^45^.

To identify gene expression patterns in the DEGs, a BLASTx homology search was conducted against the NCBI nonredundant nucleotide database (NT; http://www.ncbi.nlm.nih.gov/blast/db) and nonredundant protein database (NR; http://www.ncbi.nlm.nih.gov/blast/db) to search for archived unigenes from public databases including Swiss-Prot (http://ebi.ac.uk/interpro), KEGG (KEGG; http://www.genome.jp/kegg), and COG of Protein (http://www.ncbi.nlm.nih.gov/COG). Functional classification of the unigenes’ GO (http://www.geneontology.org/) was performed by Blast2GO^46^, and InterProScan5^47^ was used for InterPro annotation. The COG classification was performed against the COG database, and pathway analysis was performed using the KEGG annotation service.

### Total RNA extraction and cDNA synthesis

Total RNA was separately extracted from each 0.1 g sample (fresh weight) using the RNeasy Plant Mini Kit (Qiagen, Hilden, Germany) and then treated with DNase (TURBO DNA-free TM kit, Invitrogen, Carlsbad, USA) to remove genomic DNA contamination. Purity and concentration of RNA samples were calculated by GeneQuant pro spectrophotometer (UK) and the integrity of RNA samples was checked by using agarose gel electrophoresis. RNA samples displayed good quality with A260/A280 ratios ranging from 1.9 to 2.1 were used in the present study. Then, the first-stand complementary DNA (cDNA) was synthesized from 300 ng of total RNA in a volume of 20 μL with PrimeScriptTM 1st strand cDNA Synthesis Kit (TaKaRa Bio, Kusatsu, Japan) according to the manufacturer’s instructions. The cDNA was diluted 20 times before being used as templates in quantitative real-time PCR.

### Validation of the RNA sequence analyses by qRT-PCR

The relative expression levels of genes listed in Supplementary Table 2 in the thallus, conchosporangium, and conchocelis stages were measured by fluorescence qRT-PCR. The qRT-PCR was performed in triplicate for each sample with an ABI 7300 real-time PCR detection system using the SYBR Premix Ex Taq GC (Perfect Real Time) kit (TaKaRa, Japan). The total reaction volume was 20 μL, containing 10 μL of 2 × SYBR Premix Ex Taq GC solution, 0.4 μL of ROX reference dye, 0.4 μL (10 μM) of each primer, 2 μL of the diluted cDNA mix, and 6.8 μL of RNA-free water. The 18S rRNA gene was used as an internal control. The primer sequences are shown in Supplementary Tables 1 and 3. The thermal profile for the qRT-PCR was 95 °C for 30 s, followed by 40 cycles of 95 °C for 5 s and 60 °C for 32 s. Dissociation curve analysis of the amplicons (95 °C for 15 s, 60 °C for 1 min, and 95 °C for 15 s) was performed at the end of each PCR reaction to confirm that only one specific PCR product was amplified and detected. After PCR, the data were analyzed with the ABI optical system software. To maintain consistency, the baseline was set automatically by the software.

### Phylogenetic analysis

Amino acid sequences of TALE-HD proteins used for the phylogenetic analysis were obtained from GenBank, genome and EST databases, and our unpublished transcriptome analyses. These are mentioned in the legend to Fig. 4 with their accession numbers or gene IDs. An unrooted neighbor-joining phylogenetic tree was constructed with 1000 replicates of bootstrap in the MEGA 8.0 software (https://www.megasoftware.net) using ClustalW to align the TALE-HD amino acid sequences. Databases: *Arabidopsis thaliana*, https://www.arabidopsis.org; *Physcomitrella patens*, https://genome.jgi.doe.gov/Phypa1_1/Phypa1_1.home.html; *Porphyra umbilicalis*, https://phytozome.jgi.doe.gov/pz/portal.html#; *Porphyra purpurea*, http://dbdata.rutgers.edu/nori/; and *Cyanidioschyzon merolae*, http://merolae.biol.s.u-tokyo.ac.jp.

### Artificial release of single cells from conchosporangia

Growing conchosporangia were isolated from culture under an Olympus IX73 light microscope (Olympus, Tokyo, Japan) and fragmented by chopping randomly with a single-edged razor blade on a glass slide. After returning the chopped fragments to the medium, one- to five-celled fragments with cell walls were collected under a light microscope, and individual fragments were transferred separately into a single well of a 96-well microplate (Falcon Microtest 96, Becton Dickinson Labware, UK). Since some spore-like single cells were obtained by discharge from the cell wall skeleton after incubation for 2 or 4 days (unpublished observation), development of these spore-like single cells was monitored daily under a light microscope.

### Statistics and reproducibility

All data of qRT-PCR analysis are given as mean ± SE in terms of relative mRNA expression (*n* = 3). One-way ANOVA was followed by the Tukey–Kramer test for multiple comparisons, for which differences were analyzed by applying a cutoff value of *p* < 0.05.

### Reporting summary

Further information on research design is available in the Nature Research Reporting Summary linked to this article.

## Supplementary information

Supplementary Data 1

Reporting Summary

Description of Additional Supplementary Items

Supplementary Information

## Data Availability

Nucleotide sequences of unigenes encoding PyKOX, PyBELL1, and PyBELL2 were deposited as GenBank accession numbers MK629536, MN070241, and MN070242, respectively. RNA-seq data have been deposited to the NCBI Short Read Archive with accession number PRJNA554402. Source data for box plots in Figs. 1–3 and Supplementary Fig. 6 are available as Supplementary Data 1. Any other datasets generated during and/or analyzed during the current study are available from the corresponding author on reasonable request.
