# Supplementary material for: A unique life cycle transition in the red seaweed Pyropia yezoensis depends on apospory
Source: Commun Biol. 2019 Aug 7;2:299. doi: 10.1038/s42003-019-0549-5 (PMC6685973; doi:10.1038/s42003-019-0549-5)
Supplement: Supplementary file 3 — Description of Additional Supplementary Items [file 42003_2019_549_MOESM3_ESM.docx]

**Description of Additional Supplementary Items**

**Supplementary Data 1:** Source data for the box plots shown in Figures 1-3 and Supplementary Figure 6.
