# Supplementary material for: A unique life cycle transition in the red seaweed Pyropia yezoensis depends on apospory
Source: Commun Biol. 2019 Aug 7;2:299. doi: 10.1038/s42003-019-0549-5 (PMC6685973; doi:10.1038/s42003-019-0549-5)
Supplement: Supplementary file 4 — Supplementary Information [file 42003_2019_549_MOESM4_ESM.pdf]

Supplementary Figures

Supplementary Fig. 1. Length distribution of all unigenes mapped from the contigs of the comparative transcriptome analysis.

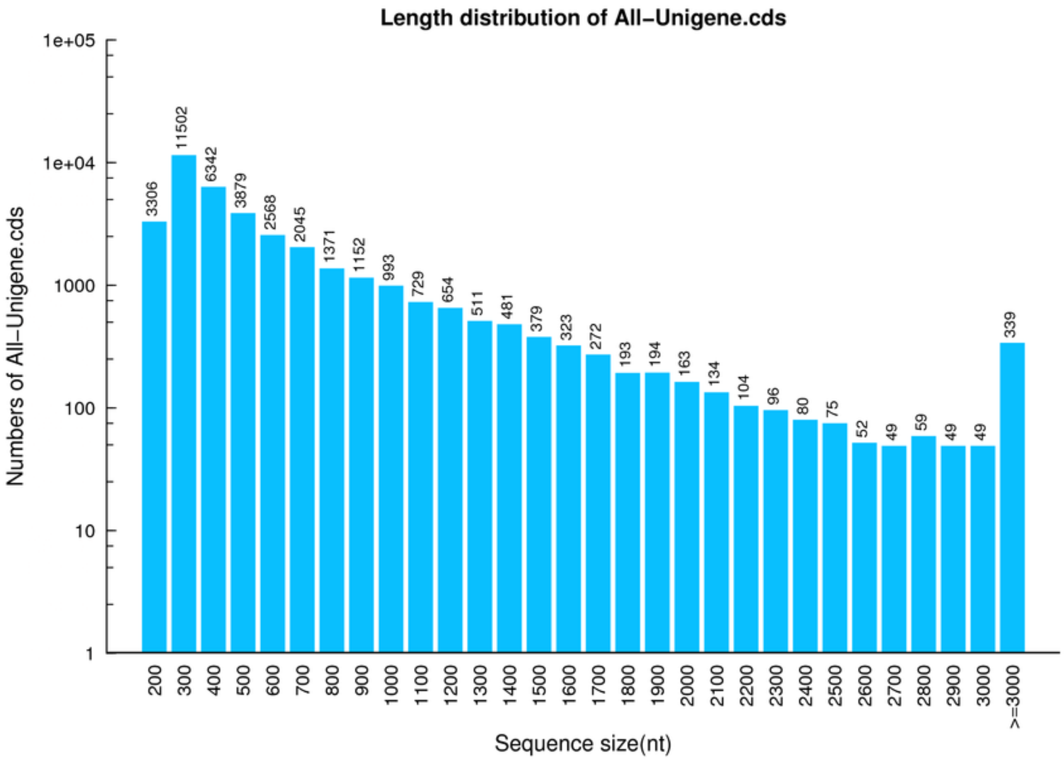

Supplementary Fig. 2. (A) Venn diagram of annotated unigenes using established databases. (B) Species distribution of all unigenes identified in three life cycle stages.

A

B

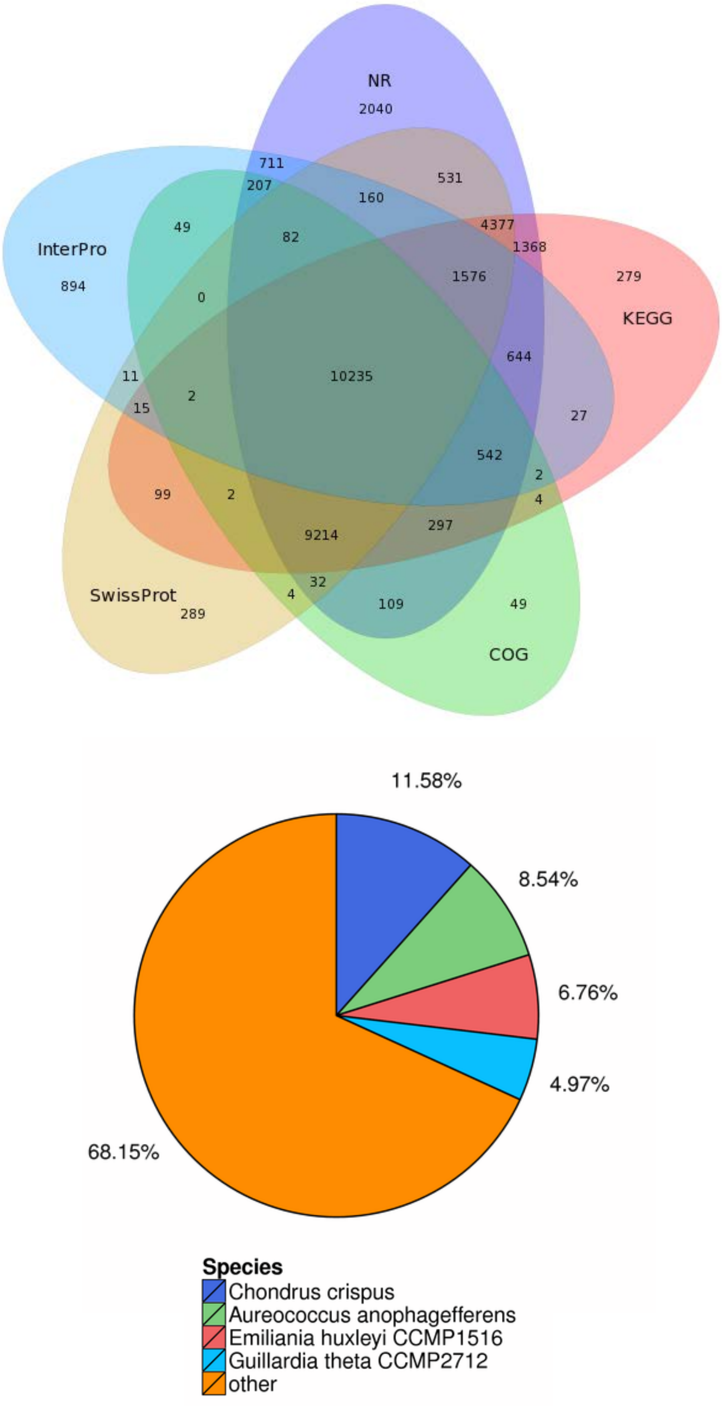

Supplementary Fig. 3. Histogram presentation of clusters of orthologous groups (COG) functional classification of assembled unigenes mapped from the contigs of the comparative transcriptome analysis.

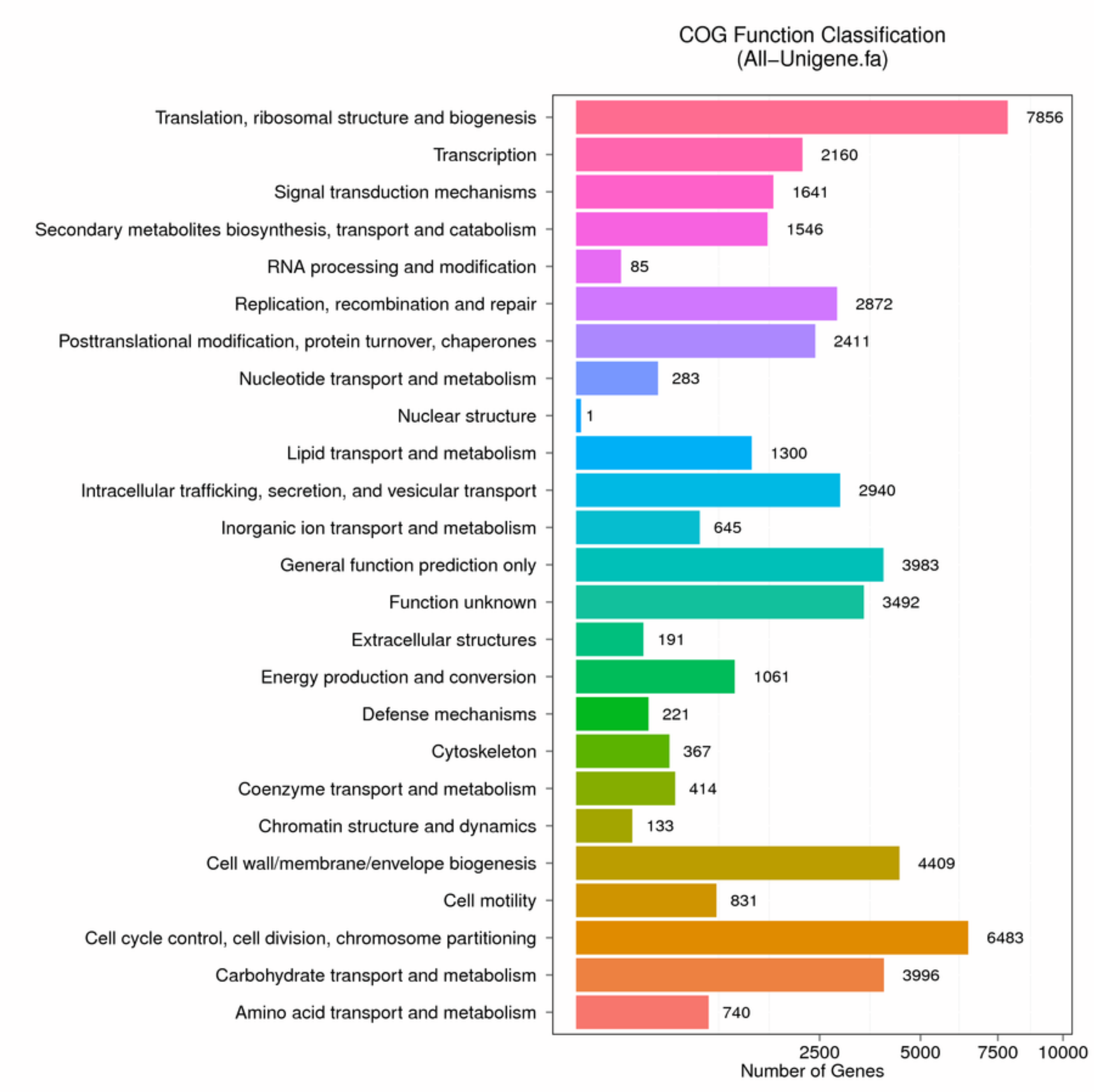

Supplementary Fig. 4. Histogram presentation of the Gene Ontology (GO) classification of assembled unigenes mapped from the contigs of the comparative transcriptome analysis.

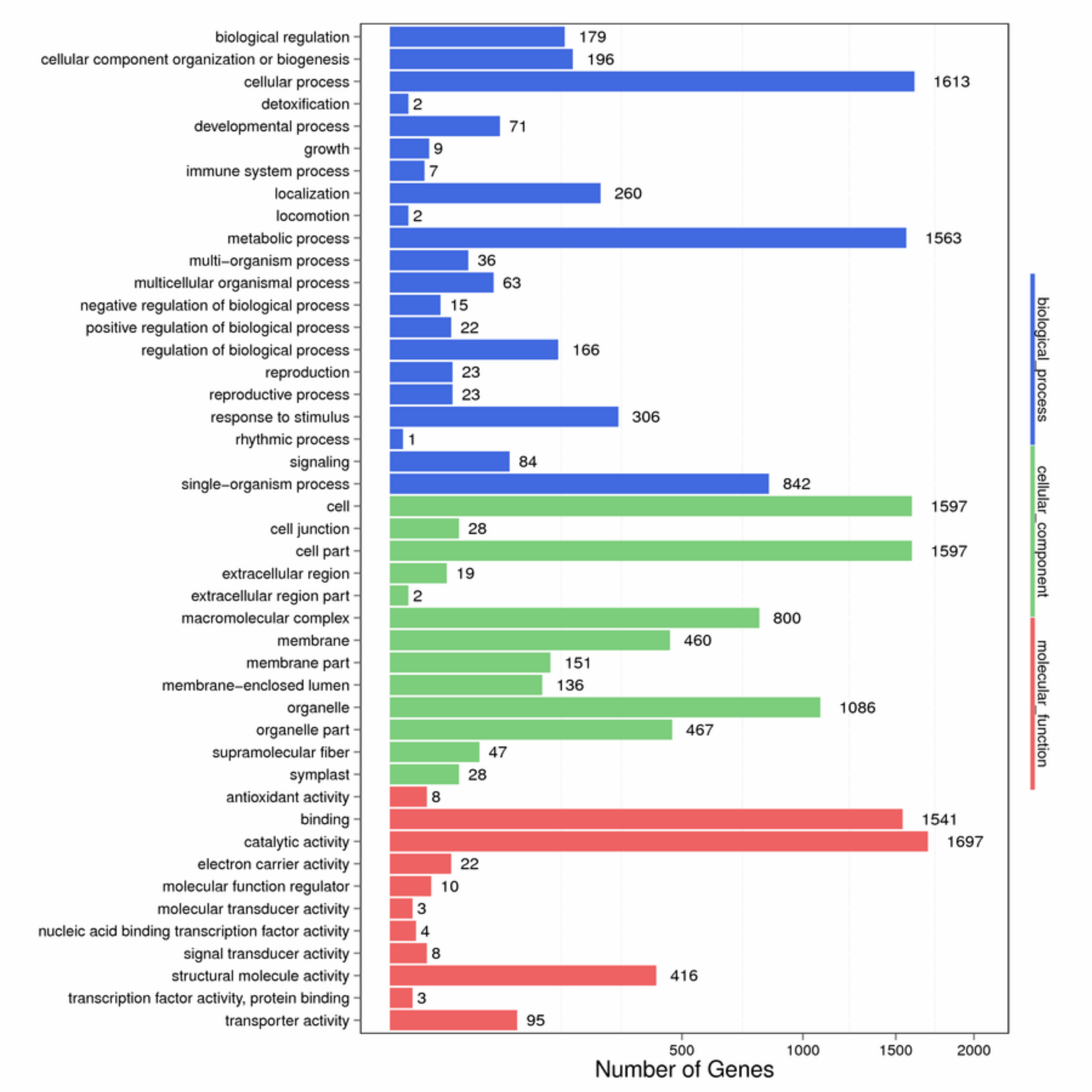

Supplementary Fig. 5. Histogram presentation of the Kyoto Encyclopedia of Genes and Genomes (KEGG) metabolic pathway annotation of assembled unigenes mapped from the contigs of the comparative transcriptome analysis.

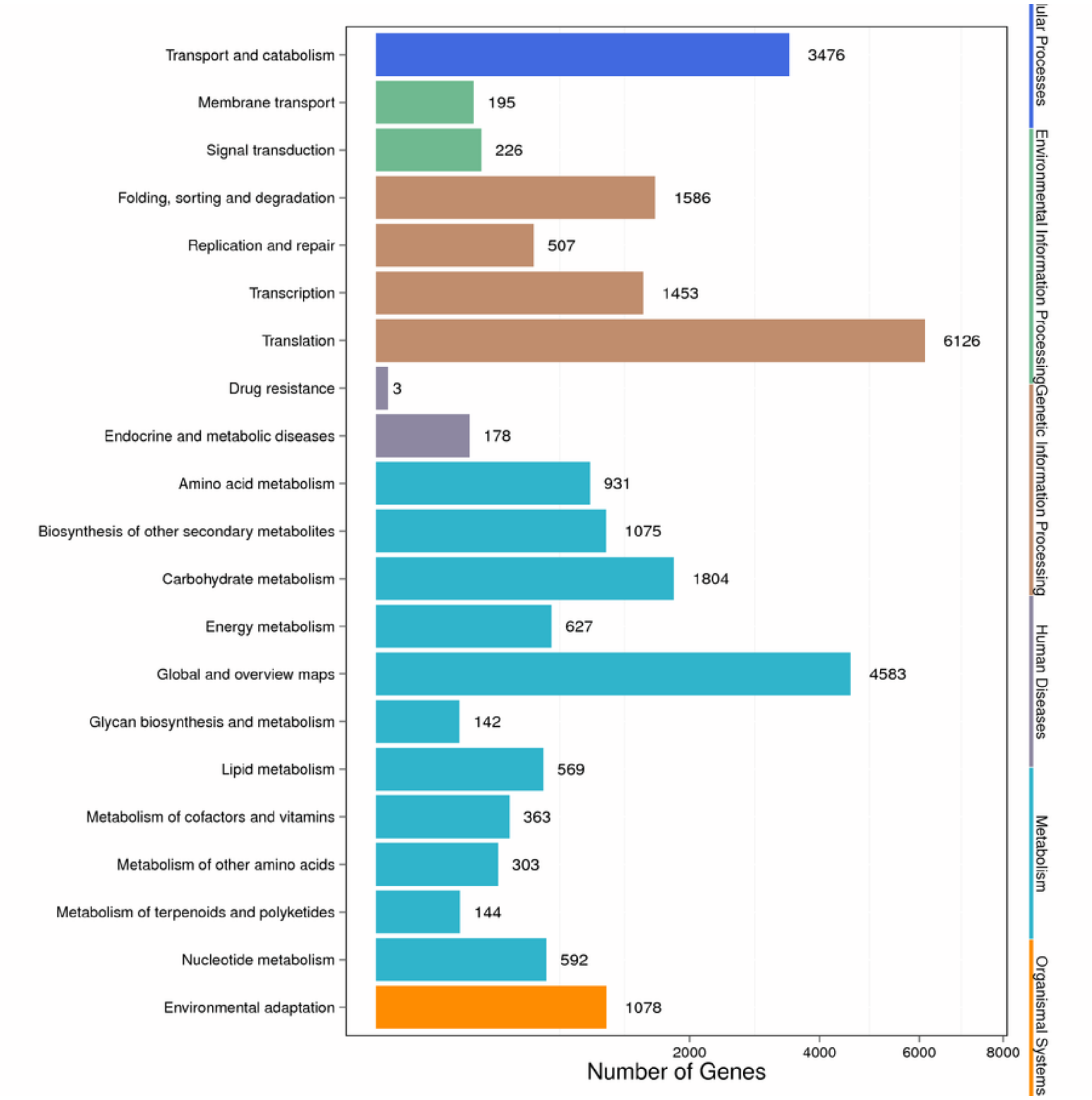

Supplementary Fig. 6. Box plots showing quantitative RT-PCR validation analysis of expression of selected genes.

Conchocelis-specific unigenes (SS3, SS4 and SS5) were selected from Table S2. The ratio as values indicated in the Y axis refers to the relative expression levels of genes among the three life cycle stages, thallus (T), conchosporangium (CS) or conchocelis (CC). The *18S* rRNA transcript was quantified as an internal reference. Alphabetical characters denote significant differences in expression levels among three life cycle stages from triplicated experiments as defined by the Tukey test ($p < 0.05$) in one-way ANOVA.

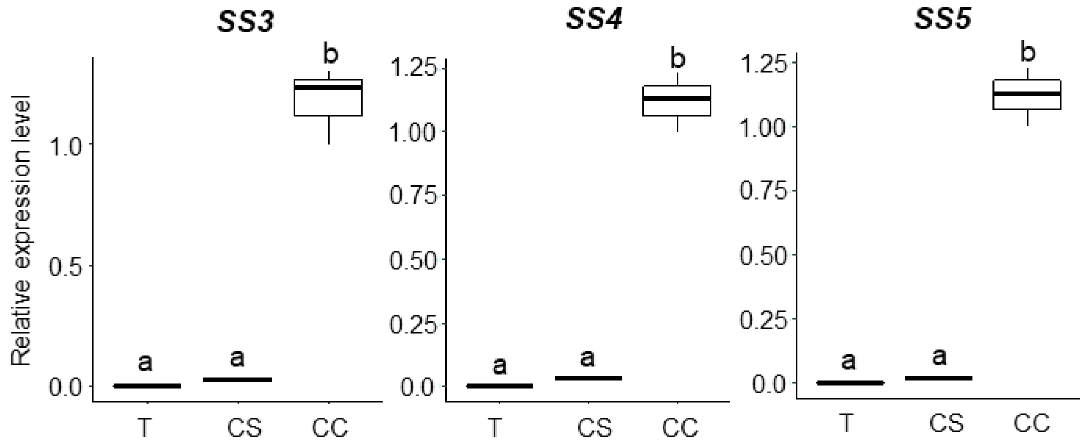

Supplementary Fig. 7. Partial amino acid sequences of the TALE-HD ptoteins from *Pyropia yezoensis*.

The conserved homeobox KN domain, which had been identified in eukaryotic TALE-HD proteins like KNOXs, is highlighted by yellow .

PyKNOX (Contig CL1448)

MEFVSAYMALMDVYEARCEALHASVDAACDALVAQSRIPELAEDGLGGGWGVGDRRGDG
SRGGRPDGRREEGGGGGKDSNRAANGDGDEGDGGGGSGSGDDDEEEDLEVLQRRVRRKF
VAAMEQLQCSRLPKRRRGNLPKEATAVFRSWFAANLDHPYPTDAVKADLSRATGTGVAQ
VSNWLINYRKRVWKPALQEHAASTGEGGAAKNAPAGGEAAAED

PyBELL1 (Contig CL1176)

SPTSAAFSAAAAAAAAAPTRRGALSARANCYLRLWLFDHFLSPYPSEADKARLAARAGL
SLVQVNNWFINARVRLWKPTVDALHTGGADTGAAAAAAAAAA

PyBELL2 (Unigene 19722)

AVSVPSHFEVPTSAFVNASSPTGSAGSAAGSQRHQSRSSSAAKGRQLPEWSVHILKEWL
LSQEHFDYPWPTPEEKTELAARAGIDERQLGIWLTNARKRLWMPLRRRQGLPIPRYA

Supplementary Fig. 8. Tip growth of the conchocelis of *Pyropia yezoensis*.
During growth of the conchocelis, only the tip cell (conchocelis stem cell) has cell division activity and can-produces a stem cell located at the tip and a downstream adjacent non-dividing cell by asymmetrical cell division, which is performed once a day. Arrows indicate the positions of the cell walls.

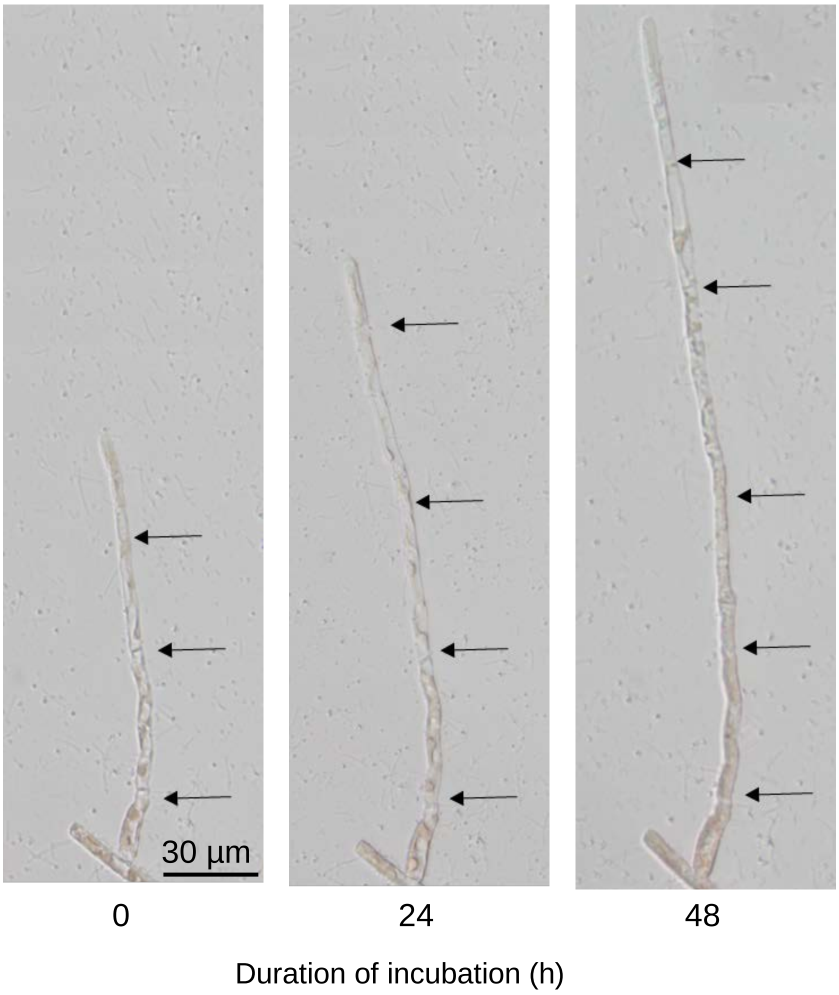

Supplementary Tables

Supplementary Table 1. Primers used in qRT-PCR for previously identified thallus- and conchocelis-specific genes.

Gene	Direction	Sequence (5' to 3')	Accession no.
PyKPA1	Forward	TACTCTTACGCAGAACCGGATGA	AB689868.1
	Reverse	AGGTCGGTGAGTCAAAGTCGAA	
PyKPA2	Forward	GCCTCATCTTCGACAATCTCAA	AB686252.1
	Reverse	CAACCAGGATGAAGGTCAGGAA	
PyAly	Forward	AAGGACCTGCGGAATACGA	AB512414.1
	Reverse	CGGGTTCCCAACACGATC	
PyDUR3.3	Forward	GGAACTTCTTCCTCGACCCTC	AB931115.1
	Reverse	ACAATAACATAGCCGACCCACTT	
PyBPO	Forward	GCTCGCCGCTAACATCTCCA	AB775530
	Reverse	GCACGGTCATACTCCAGTTCTCCC	
PyAMT1	Forward	GCACATTCTGTGGTTTGG	AB931116
	Reverse	CCGTGTTCGAGATACCCAGAGT	

Supplementary Table 2. List of top ten stage-specific and stage-dominant unigenes.

Unigene ID	Number of transcripts T	CS	CC	Top BLAST match to Nr database
**Thallus-specific unigenes**				
CL663.Contig2_All	14040.5	0.5	0.3	hypothetical protein AURANDRAFT_71560, partial [Aureococcus anophagefferens]
Unigene18725_All	4537.9	0.1	0	unnamed protein product [Chondrus crispus]
CL2220.Contig2_All	3069.3	0.4	0.3	Serine protease-like protein[Porphyra purpurea]
Unigene20936_All	1515.5	0.2	0	hypothetical protein VOLCADRAFT_104860 [Volvox carteri f. nagariensis]
Unigene8472_All	1232.9	1.1	0.3	hypothetical protein SORBI_002G360300, partial [Sorghum bicolor]
CL128.Contig1_All	1126.1	0.3	0.2	hypothetical protein AURANDRAFT_71291, partial [Aureococcus anophagefferens]
Unigene23943_All	1003.1	0.7	0.6	metalloproteinase, extracellular matrix glycoprotein VMP22 [Volvox carteri f. nagariensis]
Unigene23256_All	682.5	0.3	0	HAL3-like protein [Klebsormidium flaccidum]
Unigene18659_All	612.1	0.2	0	hypothetical protein AURANDRAFT_71601, partial [Aureococcus anophagefferens]
Unigene19608_All	475.7	0.3	0.2	NA
**Conchosporangium-specific unigenes**				
Unigene10116_All (CC4)	0	798.1	70.3	putative GRP family [Medicago truncatula]
Unigene4001_All	0.6	619.4	1.56	PREDICTED: formin-like protein 20 [Cucumis sativus]
Unigene16818_All	1.5	492.3	1.4	hypothetical protein VOLCADRAFT_94929 [Volvox carteri f. nagariensis]
Unigene34638_All	0.9	362.1	0.6	hypothetical protein AURANDRAFT_70714, partial [Aureococcus anophagefferens]
Unigene39536_All	0	351.6	0.8	NA
CL2047.Contig4_All	0	243.8	0	predicted protein [Micromonas sp. RCC299]
Unigene820_All	1.6	238.1	1.8	hypothetical protein EMIHUDRAFT_122496, partial [Emiliania huxleyi CCMP1516]
Unigene42008_All	1.2	201.2	1.9	hypothetical protein Gasu_15300 [Galdieria sulphuraria]
CL2047.Contig1_All	0	193.7	0	PREDICTED: keratin-associated protein 6-2-like [Oryza brachyantha]
Unigene41137_All	0	181.6	0	Rho GTPase-activating protein domain [Ostreococcus tauri]

Unigene ID	Number of transcripts			Top BLAST match to Nr database
	T	CS	CC	
**Conchocelis-specific unigenes**				
CL1600.Contig1_All (SS3)	0.1	4.9	1341.3	plastid oxygen-evolving enhancer 1 precursor [Pyropia yezoensis]
CL106.Contig1_All (SS4)	0.4	0.8	1155.9	unnamed protein product [Chondrus crispus]
Unigene17016_All (SS5)	0.4	0.8	1072.1	pherophorin-dz1 protein [Volvox carteri f. nagariensis]
Unigene3938_All	0.2	1.4	533.6	unnamed protein product [Chondrus crispus]
Unigene5518_All	0.	1.5	305.8	hypothetical protein AURANDRAFT_71291, partial [Aureococcus anophagefferens]
Unigene27784_All	0	0	296.2	hypothetical protein VIGAN_UM154600, partial [Vigna angularis var. angularis]
Unigene29424_All	0	0	275.9	PREDICTED: uncharacterized protein LOC103933723 [Pyrus x bretschneideri]
Unigene25218_All	0	0	247.7	hypothetical protein VOLCADRAFT_63308, partial [Volvox carteri f. nagariensis]
Unigene25751_All	0	0.7	210.2	pathogenesis-related protein 1-like protein [Volvox carteri f. nagariensis]
Unigene29053_All	0	0.7	193.2	hypothetical protein EMIHUDRAFT_101890 [Emiliania huxleyi CCMP1516]
**Thallus-baiased unigenes**				
Unigene5123_All	21425.4	68.5	0.1	basic proline-rich protein-like isoform X1 [Solanum pennellii]
Unigene10550_All	17389.8	59.5	0	hypothetical protein EMIHUDRAFT_462069 [Emiliania huxleyi CCMP1516]
Unigene10937_All	12670.3	216.3	1.6	hypothetical protein VOLCADRAFT_104860 [Volvox carteri f. nagariensis]
Unigene16775_All	7013.2	189.2	0.4	extensin-like [Setaria italica]
Unigene10222_All	6661.0	62.5	4.0	PREDICTED: extensin-like [Setaria italica]
Unigene17150_All	4028.5	38.8	0.8	NA
CL263.Contig1_All (GC5)	2170.8	91.7	835.2	elongation factor-1a [Pyropia yezoensis]
Unigene18058_All (GC6)	2021.7	213.3	767.5	elongation factor-1a [Pyropia yezoensis]
Unigene14502_All	1948.2	27.7	2.2	unnamed protein product [Chondrus crispus]
Unigene14727_All (GC2)	1106.8	54.1	54.7	putative S-adenosylmethionine synthetase [Pyropia yezoensis]
**Conchocelis-baiased unigenes**				
CL1691.Contig1_All	0.1	3160.1	4411.0	hypothetical protein AURANDRAFT_71560, partial [Aureococcus anophagefferens]

Unigene ID	Number of transcripts			Top BLAST match to Nr database
	T	CS	CC	
CL42.Contig2_All (SC1)	0.4	420.6	4331.6	NA
Unigene10689_All (SC2)	0.3	388.4	3012.4	hypothetical protein GUITHDRAFT_119564 [Guillardia theta CCMP2712]
Unigene3873_All	1.1	289.7	2022.3	Na+-ATPase [Pyropia yezoensis]
Unigene5172_All	0	118.5	2015.7	hypothetical protein VOLCADRAFT_99506 [Volvox carteri f. nagariensis]
CL118.Contig2_All	0	215.0	1797.8	photosystem II protein M [Cyanidioschyzon merolae strain 10D]
Unigene10765_All	0	715.9	1769.8	PREDICTED: extensin-like [Setaria italica]
Unigene12534_All	0	492.8	1754.1	unknown [Zea mays]
CL1649.Contig2_All	0	153.5	1406.2	V-type H(+)-translocating pyrophosphatase [Bathycoccus prasinos]
Unigene1691_All	0.1	150.9	1109.3	hypothetical protein GUITHDRAFT_119564 [Guillardia theta CCMP2712]

Gene names of unigenes employed for qPCR as shown in Fig. 2d and Supplementary Fig. 1 are indicated under the IDs of the unigenes (compare with Supplementary Table 3). The number of transcripts are represented by FRKM values obtained from the comparative transcriptome analysis. T, Thallus; CS, conchosporangium; CC, conchocelis. NA, not annotated.

Supplementary Table 3. Primers used in qRT-PCR for selected unigenes and the *PyKNOX* gene.

Gene Name	Unigene ID	Direction	Sequence (5' to 3')
SS3	CL1600.Contig1_All	Forward	GCGGAGAATGCCCTCAAT
		Reverse	CCCCCTGTCACCCAACAA
SS4	CL106.Contig1_All	Forward	GTCCCATCGGTCAAAGCG
		Reverse	GGGTGGTGGAGGGCAGCA
SS5	Unigene17016_All	Forward	CCCCTCCCGTCTTCCTCG
		Reverse	TGCTCCTTCCGTTCCCAG
CC4	Unigene10116_All	Forward	GTCAACGAGCGAGGGAGA
		Reverse	GTGCGGCAGTCAAGGAAT
CC3	Unigene914_All	Forward	GAGCGGAAAGCATGAGAA
		Reverse	CCAAGAAGGCGGTAGTAG
GC3	Unigene11848_All	Forward	TTTTCCCCCCAGCAACTA
		Reverse	CCGCACACAGCATACCAA
SC1	CL42.Contig2_All	Forward	AACAAAGAACCCGCCCAG
		Reverse	CCCACCTTCCGTCACCAT
GC5	CL263.Contig1_All	Forward	TGCTGCTTCTCCTTCCCC
		Reverse	CACACGCATCCCGCTATC
GC6	Unigene18058_All	Forward	TGCTGCTTCTCCTTCCCCA
		Reverse	ATCGTACCCGTCTTCCCCG
GC1	Unigene15445_All	Forward	GCAGATACCCTCCTTCCG
		Reverse	CGAGCTTCATGCCCAGTC
SC2	Unigene10689_All	Forward	CCAGGCGGTGTTGTGGTA
		Reverse	GGAGAGACGGAGGAGGAA
GC2	Unigene14727_All	Forward	GCAGATACCCTCCTTCCG
		Reverse	CGAGCTTCATGCCCAGTC
GC4	Unigene7445_All	Forward	CGACCCCGCACGATACGA
		Reverse	ATGAACAGAGCAGCCCCC
PyKNOX	CL1448	Forward	TGTTTCGCTCGTGGTTTGCC
		Reverse	TTCCATACCCGCTTGCGATA GTT
